# Regulation of endogenous retroviruses in murine embryonic stem cells and early embryos

**DOI:** 10.1093/jmcb/mjad052

**Published:** 2023-08-21

**Authors:** Xinyi Lu

**Affiliations:** State Key Laboratory of Medicinal Chemical Biology, Nankai University, Tianjin 300350, China

**Keywords:** endogenous retrovirus, embryonic stem cell, epigenetic regulation, transcriptional regulation, post-transcriptional regulation

## Abstract

Endogenous retroviruses (ERVs) are important components of transposable elements that constitute ∼40% of the mouse genome. ERVs exhibit dynamic expression patterns during early embryonic development and are engaged in numerous biological processes. Therefore, ERV expression must be closely monitored in cells. Most studies have focused on the regulation of ERV expression in mouse embryonic stem cells (ESCs) and during early embryonic development. This review touches on the classification, expression, and functions of ERVs in mouse ESCs and early embryos and mainly discusses ERV modulation strategies from the perspectives of transcription, epigenetic modification, nucleosome/chromatin assembly, and post-transcriptional control.

## Introduction

The majority of mammalian genomes consist of miscellaneous repetitive elements (McPherson et al., 2001; Waterston et al., 2002). Among these repetitive sequences, endogenous retroviruses (ERVs), also known as long terminal repeat (LTR) retrotransposons, account for ∼8%–10% of the human and mouse genomes (McPherson et al., 2001; Waterston et al., 2002). ERVs are transposable elements (TEs) that exhibit sequence similarity to retroviruses (Jern and Coffin, 2008) and may originate from exogenous retroviruses (Johnson, 2019). Hundreds of millions of years ago, exogenous retroviruses infected the germ cells of mammalian ancestors and integrated into the host genome (Vogt, 1997). They were transmitted to the germ cells of the offspring and eventually became part of the host genome. During evolution, the integrated retroviruses accumulated mutations and gradually lost their protein-coding potential along with their ability to reproduce viruses (Boeke and Stoye, 1997), thus becoming ERVs nowadays. Nevertheless, ERVs still retain key characteristics of retroviruses. Both mammalian ERVs and exogenous retroviruses phylogenetically belong to the *Retroviridae* family (Xiong and Eickbush, 1990), with the genome structure similar to that of other members of the family. ERVs consist of two LTR regions, a primer binding site (PBS), and genes encoding *gag, pol*, and *env*. The LTRs drive the transcription of ERVs, while the PBS region serves as a recognition site for reverse transcriptase (Coffin et al., 1997). Based on their resemblance to retroviruses (Tristem, 2000), ERVs are divided into three classes: ERV I (gamma- and epsilon-retroviruses), ERV II (lentivirus, alpha-, beta-, and delta-retroviruses), and ERV III (spuma-retrovirus-like) (Table 1; Benit et al., 1999; Gifford et al., 2005; Jern et al., 2005). The majority of ERVs are silenced and maintain neutrality toward the host genome (Jern and Coffin, 2008; Mager and Stoye, 2015). However, given their retroviral origin, ERVs are dynamically expressed during embryonic development and in pluripotent cells. Their activities contribute to the shaping of host gene expression and the organization of host genome structure. Pluripotent cells, such as embryonic stem cells (ESCs) and induced pluripotent stem cells, manifest a unique ability to silence retroviruses (Feuer et al., 1989; Maherali et al., 2007; Wernig et al., 2007; Wolf and Goff, 2007). In view of the close relationship between ERVs and retroviruses, revisiting ERV silencing mechanisms also enables us to understand the nature of retroviral silencing in ESCs. This review summarizes the current advances in understanding the regulation of ERV expression from the perspectives of transcription, epigenetics, and post-transcription in ESCs and early embryos. The expression and functional intersections between ERVs and developmental potency are also briefly reviewed.

**Table 1:** Classification of ERVs and members in each class.

Class	Sub-class	Groups
ERV I (gamma- and epsilon-retroviruses)	–	RLTR1B, RLTR6, MMVL30-int, MuLV
ERV II (lentivirus; alpha-, beta-, and delta-retroviruses)	ERVK	IAPEz, MMERVK10C, MMETn, ETnERV, ETnERV3, MusD, RLTR10B, RLTR45-int, RLTR9, RLTR10, RLTR13D, RMER19B
ERV III (spuma-retrovirus-like)	ERVL	MERVL, MT2, MT2B
	ERVL-MaLR	MTA, MTB, MTC, ORR1A0, ORR1A1, ORR1A3, ORR1D2

## Expression and functions of ERVs in mouse ESCs and early embryos

ERVs are exquisitely regulated during development and in ESCs. In mouse preimplantation embryos, each developmental stage displays distinct ERVs. Mouse transcript A (MTA), an ERV III-ERVL-MaLR group member, is present in oocytes and zygotes (Zhang et al., 2019). An ERV III-ERVL group member (MERVL) and ERV III-ERVL-MaLR group members (ORR1A0, ORR1A1, and ORR1A3) are specifically switched on in 2-cell and 4-cell embryos (Zhang et al., 2019). The 8-cell and morula embryos are characterized by the presence of the ERVK family members RLTR45-int, MMETn, and ETnERV (Zhang et al., 2019). Naïve ESCs derived from the inner cell mass (ICM) of mouse blastocyst exhibit high expression levels of long interspersed nuclear element-1 (LINE1) and RLTR1B, whereas intracisternal A particles (IAPs), MMETn, and ETnERV3 are more enriched in primed ESCs, which are derived from post-implantation epiblasts (Fu et al., 2021). ERVs are also heterogeneously expressed in ESCs, as seen in the case of MERVL. MERVL is activated in 1%–5% of mouse ESC population, referred to as 2-cell-like cells, which share transcriptome patterns similar to those of 2-cell embryos and possess the capacity to become ICM cells and trophectodermal cells (Macfarlan et al., 2012).

The dynamic activation of ERVs allows them to fulfill stage-specific functions during development. A pivotal role of ERVs is to act as *cis*-regulatory elements. ERVs are associated with transcription factors in ESCs. For example, Oct4 and Sox2 are frequently co-localized with ERVK group members in mouse ESCs (Bourque et al., 2008; Kunarso et al., 2010). The recognition of ERVs by transcription factors suggests that ERVs can serve as promoters or enhancers (Sundaram et al., 2017). Notably, RLTR13D6 and RLTR9 are bound by key pluripotency factors (Nanog and Oct4) and serve as enhancers to govern gene expression in mouse ESCs (Sundaram et al., 2017; Todd et al., 2019). An ORR1D2 locus that is bound by Oct4 and Sox2 can serve as a promoter to drive the expression of a retrotransposon-derived lncRNA, *Lx8-SINE B2*, which is critical for ESC metabolism (Chen et al., 2021b, 2022). In mouse oocytes, intronic MTC drives the expression of the *Dicer* isoform (Flemr et al., 2013). Additionally, MERVL genomic loci function as alternative promoters and enhancers to allow transcription of 2-cell genes (Macfarlan et al., 2012; Zhang et al., 2019; Chen et al., 2020). Moreover, CRISPR activation (CRISPRa) of MERVL facilitates the conversion of ESCs into 2-cell-like cells (Yang et al., 2020). Similarly, the activation of the germline-specific ERV RTLR10B by CRISPRa triggers the expression of adjacent Tdrd3 in mouse ESCs (Sakashita et al., 2020). These breakthroughs underscore the role of ERVs as enhancers and promoters to regulate gene expression during early development and in ESCs.

In addition to working as regulatory elements, ERVs are capable of forming chimeric transcripts with both non-coding and coding genes. ERVs are major contributors to long non-coding RNAs (lncRNAs) (Kapusta et al., 2013). A multitude of ESC-specific lncRNAs, termed non-annotated stem transcripts (NASTs), contain ERVK and ERVL-MaLR sequences (Fort et al., 2014). Depletion of certain NASTs impacts ESC pluripotency (Fort et al., 2014). ERVs merge with protein-coding genes throughout mouse preimplantation development (Peaston et al., 2004; Macfarlan et al., 2012). ERVs belonging to ERVL-MaLR fuse with coding genes such as *Zfp277* and *Spin*, generating chimeric transcripts in oocytes and 2-cell embryos (Peaston et al., 2004). MERVLs fuse with 2-cell transcripts such as *Zfp809* and *Rbm25* (Macfarlan et al., 2012; Chen et al., 2020), while IAPs chimerize with coding transcripts (e.g. *Akap9*) in both 2-cell embryos and blastocysts (Peaston et al., 2004). Cryptic transcription plays a role in generating these chimeric transcripts. At specific genomic locations, the splice site of MTA outcompetes the LTR polyadenylation signal, connecting with the splicing acceptor site of a gene (Peaston et al., 2004). Cryptic splicing can be regulated by the histone chaperone, named facilitates chromatin transcription (FACT) (Chen et al., 2020). These discoveries illuminate the dynamic expression profile of ERVs and their essential functions during development and in stem cells. Thus, the stage-specific expression of ERVs needs to be tightly controlled to exert their functions.

## Transcriptional regulation of ERVs in ESCs and during early development

### Transcriptional activators of ERVs

**Figure 1:**
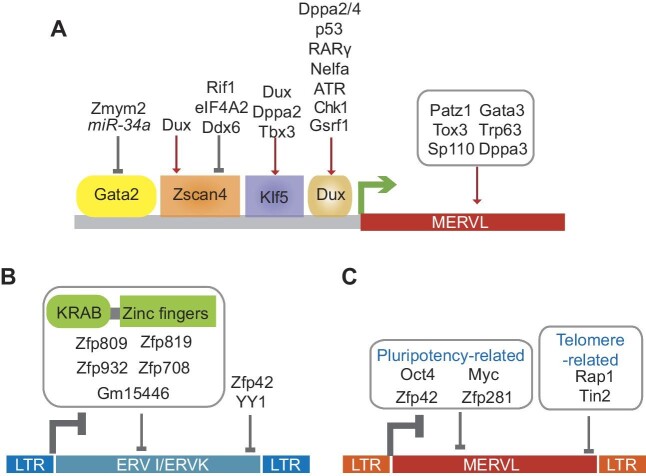
Transcriptional regulation of ERVs. (**A**) Direct transcriptional activators (Gata2, Zscan4, Klf5, and Dux) and indirect regulators of MERVL. (**B**) Transcriptional repression of ERV I/ERV II (ERVK) by KRAB-ZFPs and other transcription factors. (**C**) Transcriptional repressors of ERV III (MERVL).

To achieve temporal regulation of ERV expression during development, transcriptional activation is necessary. Studies with mouse ESCs primarily focused on the activation of MERVL due to its central role in controlling stem cell potency (Huang et al., 2017; Yang et al., 2020). Inhibition of MERVL expression with siRNA in zygote/2-cell embryos causes ∼90% of the embryos to arrest at the 2-cell stage (Huang et al., 2017). Conversely, activation of MERVL is sufficient to induce ESCs to enter a 2-cell-like state (Yang et al., 2020). Since transcription factors usually co-express with their target genes, it was expected that the transcription activators of MERVL are co-expressed with it. Gata2 and Dux were the first two transcription factors reported to directly activate MERVL (Figure 1A; Choi et al., 2017; De Iaco et al., 2017; Hendrickson et al., 2017; Whiddon et al., 2017). Similar to MERVL, *Gata2* and *Dux* are induced at the 2-cell stage during zygotic genome activation (ZGA) and subsequently inactivated during preimplantation development (Choi et al., 2017; De Iaco et al., 2017; Hendrickson et al., 2017; Whiddon et al., 2017). Dux-cluster transcription factors (Duxf3, Gm4981, Gm19459, Gm10807, and AW822073) are also critical for the surge of MERVL during ZGA (De Iaco et al., 2017; Hendrickson et al., 2017; Whiddon et al., 2017). Several other transcription factors indirectly promote MERVL expression through Dux (Figure 1A). Dppa2/4 and p53 indirectly drive MERVL expression by stimulating *Dux* expression (De Iaco et al., 2019; Eckersley-Maslin et al., 2019; Yan et al., 2019). While p53 overexpression is able to restore *Dux* levels upon *Dppa2/4* deletion, Dppa2/4 overexpression cannot rescue *Dux* expression following *p53* loss (Grow et al., 2021), suggesting that p53 lies downstream of Dppa2/4 in activating *Dux*. Moreover, the retinoic acid nuclear receptor RARγ and the elongation factor Nelfa turn on MERVL expression and 2-cell-like fate through Dux (Tagliaferri et al., 2019; Hu et al., 2020; Iturbide et al., 2021; Wang et al., 2021). The convergence of transcriptional regulators acting on *Dux* indicates an important role for *Dux* in the initiation of MERVL expression. Surprisingly, despite the potent ability of Dux to activate MERVL, its deletion only causes minor defects in ZGA in mouse embryos and does not affect mouse development; meanwhile, MERVL is only moderately disrupted and remains at high levels in 2-cell embryos lacking *Dux* (Chen and Zhang, 2019; Guo et al., 2019). These findings suggest the existence of other factors in 2-cell embryos that can sustain the expression of MERVL and support embryonic development in the absence of *Dux.*

The transcription factors Patz1, Zscan4, Gata3, Tox3, Sp110, and Trp63 were identified as potential MERVL activators in a MERVL-driven reporter screen (Figure 1A; Eckersley-Maslin et al., 2019; Alda-Catalinas et al., 2020). Among these transcription factors, Zscan4 displays direct transcriptional activation toward MERVL in ESCs (Figure 1A; Zhang et al., 2019). Zscan4 directly binds to and activates MERVL by promoting enhancer-related epigenetic modifications (Zhang et al., 2019). Depletion of *Zscan4* disrupts MERVL expression in ESCs (Zhang et al., 2019). Another transcription factor that directly activates MERVL is Klf5 (Figure 1A). Klf5 binds to and stimulates the expression of MERVL as well as other 2-cell-associated ERVs such as ORR1A0 and ORR1A1 (Kinisu et al., 2021). In addition, Klf5 confers ESCs with the bipotential ability to differentiate into both ICM and trophectodermal lineages (Kinisu et al., 2021), suggesting that the conversion of ESCs to a 2-cell-like state could be exploited as a potential way to improve the trophectodermal differentiation efficiency of ESCs. It appears that 2-cell transcription factors can regulate each other (Figure 1A). Dux recognizes the promoter of *Zscan4* and drives its transcription (Tagliaferri et al., 2019). Similarly, *Klf5* is induced by other 2-cell transcription factors, including Dux, Dppa2, and Tbx3 (Kinisu et al., 2021). Interestingly, loss of transcriptional activators of MERVL has varying effects on embryonic development. While loss of the key MERVL activator Dux does not lead to abnormalities in preimplantation development, deletion of *Gata2* causes embryo death at E10.5 (Tsai and Orkin, 1997), *Klf5* knockout leads to defects in trophectoderm lineage specification in E2.5 and E3.5 embryos (Lin et al., 2010), and disruption of *Zscan4* expression with siRNAs delays 2-cell embryo development and prevents blastocyst outgrowth (Falco et al., 2007). These findings suggest that activators of MERVL may also function at developmental stages beyond the 2-cell stage. Furthermore, several factors, including ataxia telangiectasia and Rad3-related protein (ATR)/Gsrf1 (Atashpaz et al., 2020), eIF4A2/Ddx6 (Li et al., 2022), *miR-344* (Atashpaz et al., 2020), and *miR-34a* (Choi et al., 2017), indirectly control MERVL expression by promoting or inhibiting the transcription activators of MERVL (Figure 1A). Replication stress responses mediated by ATR and Chk1 cause Gsrf1-dependent accumulation of *Dux* mRNA in 2-cell-like cells (Atashpaz et al., 2020). eIF4A2 activates translation initiation of *Ddx6* mRNA and recruits Ddx6 protein to degrade *Zscan4* mRNA (Li et al., 2022). Both *miR-344* and *miR-34* act through Gata2 in ESCs, with *miR-344* indirectly activating *Gata2* by inhibiting *Zmym2* (Atashpaz et al., 2020) and *miR-34* directly repressing *Gata2* mRNA (Choi et al., 2017). These discoveries support the idea that transcription factors work in concert with other factors to ensure the successful activation of MERVL. Although multiple factors have been implicated in the increment of ERVs, the specific mechanisms by which they cooperate to maintain ERVs are still unclear. It remains to be determined which factors are responsible for the complete activation of MERVL in 2-cell embryos and 2-cell-like cells.

### Transcriptional repressors of ERVs

The most well-known repressors of ERVs are Krüppel-associated box domain-containing zinc finger proteins (KRAB-ZFPs). During the evolution of ERVs, KRAB-ZFPs co-evolved with ERVs and mediated their silencing (Ecco et al., 2017; Bruno et al., 2019). By recognizing ERVs with their zinc finger domains, KRAB-ZFPs utilize the KRAB domain to recruit epigenetic silencers, thereby attenuating ERV expression (Peng et al., 2000; Wolfe et al., 2000). One classic example of KRAB-ZFPs in mouse ESCs is Zfp809 (Figure 1B). Zfp809 represses the ERV I family members RLTR6 and MMVL30-int by recognizing the PBS region (Wolf et al., 2015). The repressive activity of Zfp809 extends beyond ESCs, as its deletion also leads to the elevation of MMVL30-int levels in somatic tissues (Wolf et al., 2015). Due to sharing the same PBS site with MMVL30, murine leukemia virus (MuLV) is silenced by Zfp809 as well (Wolf and Goff, 2009). In view of the vital role of PBS in viral reverse transcription, the occupancy of PBS by KRAB-ZFPs makes it challenging for ERVs to evade silencing without reducing replication efficiency (Cullen and Schorn, 2020). Apart from PBS, KRAB-ZFPs can target other regions of ERVs. For instance, the ESC-specific KRAB-ZFP Zfp819 represses the 5′ LTR and *pol* of IAP and MERVL (Tan et al., 2013; Fernandes et al., 2022). A genetic screen of KRAB-ZFPs with the ERV-reporter revealed that *Zfp932* and *Gm15446* target distinct ERVK group members in ESCs by recognizing a sequence overlapping with the 3′ polypurine tract (Ecco et al., 2016). KRAB-ZFPs also contribute to the attenuation of ERVs in early embryos. Maternal Zfp708 inactivates the ERVK group member RMER19B and adjacent genes in oocytes and zygotes (Ecco et al., 2016). These studies substantiate KRAB-ZFPs as imperative transcriptional repressors of ERVs in ESCs and during early development (Figure 1B).

Interestingly, transcription factors associated with pluripotency also exhibit repressive activity toward ERVs (Figure 1C). Oct4, a core transcription factor in ESCs, shows an opposite expression pattern compared to MERVL during the transformation of ESCs into 2-cell-like cells (Macfarlan et al., 2012). In preimplantation mouse embryos, *Oct4* mRNA is present from the oocyte to the blastocyst stage (Fukuda et al., 2016). However, Oct4 protein remains cytoplasm-localized until the 8-cell stage (Fukuda et al., 2016), whereas MERVL is transcribed only in 2-cell and 4-cell embryos (Zhang et al., 2019). *Oct4* overexpression impedes the induction of MERVL in 2-cell embryos (Fukuda et al., 2016). Overexpression of nucleus-localized Oct4 in zygotes results in the inactivation of ERVs associated with ZGA, including MERVL and ORR1As, in 2-cell embryos (Fukuda et al., 2016), implicating a potential role of Oct4 in restricting MERVL. Myc and Nanog, two other pluripotency-related transcription factors, also repress MERVL in ESCs (Cartwright et al., 2005; Takahashi and Yamanaka, 2006; Smith et al., 2010; Fu et al., 2019; Zhao et al., 2022). In addition, transcription factors associated with both naïve and primed pluripotency take part in the repression of MERVL (Figure 1C). The naïve pluripotency marker Rex1 (Zfp42) suppresses MERVL, IAP, and MusD in ESCs (Guallar et al., 2012). Likewise, the primed pluripotency regulator Zfp281 represses MERVL (Fidalgo et al., 2016; Dai et al., 2017). Thus, it is likely that MERVL levels need to be restricted in both naïve and primed pluripotent stem cells. Pluripotency-related transcription factors also aid in the suppression of other ERVs. YY1 is involved in pluripotency maintenance in both ESCs and extended pluripotent stem cells (Wang et al., 2018; Dong et al., 2022), whereas it impedes the expression of MuLV in ESCs (Schlesinger et al., 2013). Together, the above studies show that the repression of ERVs and the promotion of pluripotency can be accomplished simultaneously.

Another group of ERV repressors in ESCs are telomeres and their associated proteins (Figure 1C). Mouse ESCs are characterized by their long telomeres, which are maintained by telomerase and other telomere-associated factors (Liu, 2017). Telomere shortening can activate the expression of ERVs, including MERVL, IAPEy, and ERVB3/4 (Zhao et al., 2023a). The MERVL-activating transcription factor Zscan4 is one of the factors that promote telomere lengthening through alternative extension of telomeres (Zalzman et al., 2010). In ESCs, the telomere-binding protein Rif1 prevents the expression of all three classes of ERVs (Li et al., 2017). Rif1 hinders Zscan4 activation and hyper-telomeric recombination (Dan et al., 2014). Furthermore, depletion of *Rif1* boosts the level of ERVs in ESCs lacking DNA methyltransferases (Li et al., 2017). The rise in MERVL following *Rif1* depletion can be attributed to the increment of *Zscan4*, while the upregulation of other ERVs is due to the impaired recruitment of repressive histone modifying enzymes (Ehmt2 and Suv39h1) to ERVs (Li et al., 2017). Unlike Zscan4, other proteins that help maintain telomere length are suppressors rather than activators of ERVs (Figure 1C). Telomere-associated proteins, such as Tin2 and Rap1, play a crucial role in silencing MERVL in ESCs (Barry et al., 2022; Yin et al., 2022). These findings underscore the significance of telomere length and telomere-associated proteins in the regulation of ERV expression.

In summary, transcription factors ensure the genome stability and potency of ESCs by repressing ERVs. Interestingly, the loss of a single MERVL activator does not necessarily disrupt its expression and embryonic development, while the loss of a single critical repressor is usually sufficient to activate ERVs and bias ESC fate.

## Epigenetic modification-based regulation of ERVs

### Deposition of repressive histone marks

**Figure 2:**
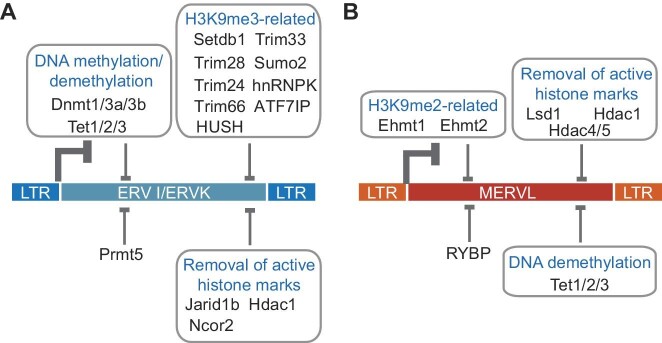
Restriction of ERVs by epigenetic modifiers. (**A**) Transcriptional repression of ERV I/ERV II (ERVK) by DNA methylation/demethylation-related enzymes, H3K9me3-related factors, and enzymes that remove active histone marks. (**B**) Transcriptional repression of ERV III (MERVL) by H3K9me2-related enzymes, enzymes that remove active histone marks, and enzymes that catalyze DNA demethylation.

Epigenetic control is at the battlefront to defend against ERVs. Epigenetic modifications stabilize the repression of ERVs resulting from transcriptional silencing (Rowe et al., 2013) and safeguard cells from the retrotransposition and replication of ERVs (Ribet et al., 2008; Maksakova et al., 2009; Macfarlan et al., 2012; Bertozzi et al., 2021). Upon recognition of ERVs, transcription factors recruit various epigenetic repressors to stably silence ERVs by inducing heterochromatin formation. KRAB-ZFPs use the KRAB domain to recruit Trim28 (also known as KAP1 or TIF1β) upon binding to ERVs through their zinc finger domains (Peng et al., 2002). Trim28 further compacts chromatin by collaborating with the nucleosome remodeling and deacetylase (NuRD) complex to remove histone acetylation. Subsequently, it recruits HP1 and Setdb1 to organize H3K9me3-marked heterochromatin (Nielsen et al., 1999; Schultz et al., 2001, 2002). Trim28 represses a wide range of ERVs, including IAPs and MERVL in ESCs (Rowe et al., 2010). Other complexes also work in conjunction with or through Trim28 to repress ERVs (Figure 2A). For example, the human silencing hub (HUSH) complex cooperates with Trim28 to silence IAPs and evolutionarily young LINE1 in naïve ESCs (Robbez-Masson et al., 2018). Sumo2 indirectly participates in the silencing of ERVs by mediating the SUMOylation of Trim28 (Yang et al., 2015). Hnrnpk promotes the SUMOylation of Trim28, which recruits Setdb1 to chromatin to repress class I and class II ERVs in ESCs (Thompson et al., 2015). In mouse embryos, Trim28 further recruits HP1γ and interacts with DNA methyltransferases to induce *de novo* methylation of ERV DNA, thereby ensuring heritable ERV silencing (Quenneville et al., 2012; Rowe et al., 2013). Besides Trim28, two other members of the TIF family, Trim24 (also known as TIF1α) and Trim33 (also known as TIF1γ), also demonstrate the ability to repress all three classes of ERVs in ESCs (Margalit et al., 2020), possibly by recruiting Hdac1, Hdac2, and HP1, as proposed in fibroblasts (Herquel et al., 2013). The fourth member of the TIF family, Trim66 (also known as TIF1δ), recognizes H3K4-K9me3 and recruits DAX1 (also known as Nr0b1) to repress Dux transcription, indirectly leading to the repression of MERVL (Zuo et al., 2022).

Epigenetic silencers display class specificity in repressing ERVs (Figure 2A and B). In ESCs, deletion of *Setdb1* activates 69 ERV subfamilies (Karimi et al., 2011). ERV I and ERV II are dominantly silenced by Setdb1 and H3K9me3 (Figure 2A; Matsui et al., 2010; Karimi et al., 2011). Although Setdb1 represses class III ERV (MERVL), it may act indirectly by repressing Dux (Wu et al., 2020). In addition, Setdb1 cooperates with other factors to repress ERVs (Figure 2A). ATF7IP retains Setdb1 in the nucleus to repress IAPs and ERVK group members in ESCs (Tsusaka et al., 2019). Suv39h1 and Suv39h2 catalyze H3K9me3 (Peters et al., 2001) and coordinate the silencing of intact ERVs in ESCs (Bulut-Karslioglu et al., 2014). On the other hand, ERV III family members are devoid of H3K9me3 but bear the mark of H3K9me2 (Maksakova et al., 2013). Both Ehmt1 (also known as GLP) and Ehmt2 (also known as G9a), which catalyze H3K9me2 (Tachibana et al., 2002), are required for ERV III silencing (Maksakova et al., 2013). The silencing and methylation of newly integrated exogenous retroviruses (e.g. MuLVs) in ESCs also depend on the presence of Ehmt1 (Leung et al., 2011). While H3K9 methylation plays an essential role, readers of H3K9 methylation are not necessary for ERV repression (Maksakova et al., 2011). However, RING1 and YY1 binding protein (RYBP), a reader of the histone mark H2AK119ub, has been found to suppress ERVs such as MERVL (Hisada et al., 2012). RYBP binds to target loci independent of H3K27me3, and its deletion does not disturb the recruitment of other polycomb repressive complex 1 (PRC1) components (Ring1B and Mel18) to their targets (Hisada et al., 2012), suggesting that RYBP may regulate MERVL expression independently of the PRC1 complex.

### DNA methylation

Dnmt-mediated DNA methylation acts as an additional mechanism to repress ERV expression (Figure 2A). In 4-cell and 8-cell mouse embryos, Dnmt1 primarily targets ERVK family members and LINE1 (Min et al., 2020). However, the majority of ERVs remain unchanged in DNA-hypomethylated ESCs carrying a triple knockout of *Dnmt1/2/3* (Tsumura et al., 2006; Karimi et al., 2011). DNA demethylation also takes place in ESCs transitioning from serum/LIF to 2i culture conditions (Leitch et al., 2013; Walter et al., 2016). ERVs (e.g. MERVL and IAPEz) exhibit a burst of transcription in the initial phase of demethylation and are later re-silenced by H3K9me3 and H3K27me3 in ESCs cultured under the 2i condition (Walter et al., 2016). H3K9me3 and DNA methylation cooperate to repress IAPs through Setdb1 (Sharif et al., 2016). The loss of *Dnmt1* permits the binding of protracted Uhrf1 (also known as NP95) to the hemi-methylated ERVK and hence disrupts ERVK silencing by Setdb1 (Sharif et al., 2016). However, in the absence of both Dnmt1 and Uhrf1, Setdb1 is able to reinstate the repression of ERVK upon the dilution of hemi-methylated DNA (Sharif et al., 2016). In addition to Setdb1, the histone arginine methyltransferase Prmt5 contributes to the silencing of IAPs in preimplantation embryos and ESCs during DNA hypomethylation (Kim et al., 2014). Furthermore, ten–eleven translocation (Tet) family proteins (Tet1, Tet2, and Tet3), which are DNA methylation hydroxylases, ensure the silencing of ERVs in the absence of DNA methylation as well (Figure 2A). Tet proteins catalyze stepwise oxidation of 5-methylcytosine (5mC) to produce 5-hydroxymethylcytosine (5hmC), 5-formylcytosine (5fC), and 5-carboxylcytosine (5caC) (Kohli and Zhang, 2013). Knockout of all three *Tet* genes (*Tet1, Tet2*, and *Tet3*) together does not alter global 5mC level but causes losses of 5hmC, 5fC, and 5caC in ESCs (Lu et al., 2014). Interestingly, all three classes of ERVs are relieved from repression upon the loss of *Tet* genes in ESCs (Lu et al., 2014). The activation of the ERV III family member MERVL can be attributed to the reduction in Trim28 binding in the absence of *Tet* genes (Lu et al., 2014), although the reasons for the activation of other ERVs remain to be elucidated. Dppa3 (also known as PGC7 or Stella) has been found to protect IAP from DNA demethylation in zygotes (Nakamura et al., 2007), while its absence in 2-cell embryos impairs MERVL activation (Huang et al., 2017). These discoveries indicate that multiple layers of regulatory mechanisms exist in addition to H3K9 methylation and DNA methylation to ensure the repression of ERVs.

### Removal of active histone marks

An alternative strategy for repressing ERVs involves the removal of active histone marks (Figure 2B). Active promoters are typically characterized by the presence of H3K4me3 (Barski et al., 2007), a histone mark whose demethylation is catalyzed by Lsd1 (also known as Kdm1a) (Shi et al., 2004). In Lsd1-deficient ESCs, the activation of ERVs such as MERVL and ORR1B1 is accompanied by increased H3K4me3 and H3K27ac as well as decreased H3K9me2 levels (Macfarlan et al., 2011). Likewise, another H3K4me2/3 demethylase, Jarid1b (also known as Kdm5b), inhibits MERVL in 2-cell embryos (Yang et al., 2022). Interestingly, despite functioning primarily as a histone demethylase, Jarid1b recruits the H3K9me3 methyltransferase Setdb1 to suppress MMVL30-int in somatic cells (Zhang et al., 2021). Upon activation, ERVs are known to be marked by H3K27ac and H3K56ac (He et al., 2019), which are targets of histone deacetylases (HDACs). Hdac1, a class I HDAC, represses RLTR45-int, ETnERV3, and MERVL in ESCs (Reichmann et al., 2012). Loss of *Ncor2*, a co-repressor of HDAC, evokes the transcription of ERVs marked by H3K9me3 and H3K56ac in ESCs (He et al., 2019). Similar to constitutively active class I HDACs, dynamically regulated class IIa HDACs, such as Hdac4 and Hdac5, also participate in the repression of MERVL in ESCs by removing H3K9ac, thereby allowing the deposition of H3K9me1 and H3K9me2 (He et al., 2019; Zhao et al., 2022). Collectively, these studies demonstrate that ERVs can be activated once the barriers against the active histone mark are removed (Figure 2A and B).

In summary, the aforementioned studies reveal that the restriction of ERVs involves multiple layers of epigenetic modifiers, both directly and indirectly. Effective control of ERVs requires a combination of the deposition of silencing epigenetic marks and the removal of active histone marks. It is important to note that different ERV loci in the same class may carry distinct epigenetic marks, highlighting the complexity of ERV regulation by chromatin modifiers. Additionally, epigenetic modifications on ERVs appear to be influenced by the surrounding chromatin context (He et al., 2019). Further investigation is warranted to elucidate intricate interplay between epigenetic modifiers and histone marks in repressing ERVs in ESCs and during development.

## Roles of nucleosome assembly and chromatin organization in the control of ERVs

### Histone variants and chaperones

**Figure 3:**
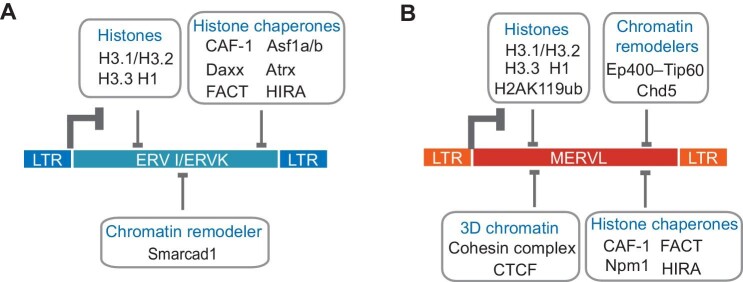
Repression of ERVs by nucleosome assembly and chromatin organization. (**A**) Transcriptional repression of ERV I/ERV II (ERVK) by histone variants, histone chaperones, and chromatin remodeler. (**B**) Transcriptional repression of ERV III (MERVL) by histones, chromatin remodelers, histone chaperones, and proteins involved in 3D chromatin organization.

In mammalian cells, the DNA genome is occluded by histones and other proteins to form chromatin. As part of genomic DNA, ERVs are also regulated by the assembly of nucleosomes and chromatin structure. A nucleosome is formed by wrapping DNA around four pairs of histones: H3, H4, H2A, and H2B. Incorporation of histone variants confers nucleosome dynamics and diversity to cope with transcriptional regulation. Both histone variants and histone chaperones, which are responsible for histone deposition, participate in the modulation of ERVs (Figure 3A). The histone chaperone chromatin assembly factor-1 (CAF-1) coordinates the deposition of histones H3.1/H3.2 and H4 during DNA synthesis (Tagami et al., 2004). CAF-1 represses IAP, LINE1, and short interspersed nuclear element (SINE) in morula embryos via H3K9me3 and H4K20me3 (Hatanaka et al., 2015). CAF-1 also chaperones the replacement of H3.3 with H3.1/H3.2 on its target retrotransposons in blastocysts (Hatanaka et al., 2015). However, CAF-1 behaves differently in ESCs and mainly impedes MERVL, MMERGLN, and MMERVK10C (Ishiuchi et al., 2015; Yang et al., 2015). Consistent with the role of CAF-1 as an H3.1 chaperone, MERVL^+^ 2-cell-like cells exhibit high mobility of H3.1 (Ishiuchi et al., 2015). These results suggest a repressive role for H3.1/H3.2 in the regulation of ERVs. In contrast, H3.3 is deposited in different regions compared to H3.1/H3.2. During preimplantation development, H3.3 is enriched in zygotic heterochromatin and then translocates to MERVL loci in early 2-cell embryos (Ishiuchi et al., 2021). H3.3 carrying H3K9me3 is loaded by the histone chaperone Atrx–Daxx to repress ERV I and ERV II in ESCs (Elsasser et al., 2015; He et al., 2015; Sadic et al., 2015). In addition to acting through H3.3, Daxx complexes with Setdb1, Trim28, and Hdac1 to repress ERVs, bypassing Atrx and H3.3 (Hoelper et al., 2017). Meanwhile, Daxx collaborates with other proteins to restrain ERVs (Desai et al., 2021; Groh et al., 2021). Morc3, a nuclear matrix-localized protein, interacts with Daxx to restrict IAPs and other ERVs through Daxx-mediated H3.3 incorporation (Desai et al., 2021; Groh et al., 2021). Loss of another H3.3 histone chaperone, the histone cell cycle regulator (HIRA) complex, activates ERV expression as well (Zhang et al., 2022). Surprisingly, Hira and Ubn2, two components of the HIRA complex, regulate distinct ERV classes (Zhang et al., 2022). Ubn2 directly represses LINE1, ERVK, and MERVL, whereas Hira primarily blocks the expression of ERVK and ERV I (Zhang et al., 2022). CAF-1 and the HIRA complex require the assistance of Asf1a and Asf1b for loading H3 histones (Tagami et al., 2004; Tang et al., 2006). Deficiency of *Asf1a* or *Asf1b* alone influences the development of preimplantation embryos (Wang et al., 2021) but not the expression of ERVs (Yang et al., 2015). However, simultaneous depletion of *Asf1a* and *Asf1b* disrupts MuLV silencing, implying complementary functions of *Asf1a* and *Asf1b* (Yang et al., 2015).

Histones H2A and H2B are integral parts of the nucleosome, along with H3 and H4. Disruption of the function of the H2A and H2B chaperone FACT activates all three classes of ERVs, with MERVL showing the most significant upregulation (Figure 3A and B; Chen et al., 2020). FACT recruits the histone deubiquitinase Usp7 to remove the active histone mark H2Bub, thereby repressing MERVL (Chen et al., 2020). Interestingly, H2A ubiquitination has the opposite effect on transcription. H2A can be ubiquitinated at K119 by the PRC1 complex, resulting in polycomb-mediated repression (Tamburri et al., 2020). H2AK119ub levels are lower in MERVL^+^ 2-cell-like cells than in ESCs (Rodriguez-Terrones et al., 2018), suggesting that MERVL activation entails H2AK119ub decrement. Supporting the role of H2AK119ub, depletion of the PRC1.6 subunit Ring1b, Pcgf6, RYBP, Mga, or Max alleviates MERVL repression in ESCs (Rodriguez-Terrones et al., 2018). In contrast, the absence of the PRC2 component EED or EZH2 does not affect MERVL expression (Rodriguez-Terrones et al., 2018), confirming the dispensable role of PRC2 and H3K27me3 in MERVL repression.

In addition to the core components of the nucleosome, linker histone H1 and its corresponding chaperone also play a role in the suppression of ERVs. Histone H1 and its variants compact the nucleosome array into heterochromatin. Recently, it was discovered that histone H1 variants and Nucleophosmin 1 (Npm1), a histone chaperone of H1, are involved in repressing ERVs in ESCs (Zhao et al., 2023b). The loss of histone H1 variants (H1.2, H1.3, and H1.4) mainly activated the expression of the ERVK family and MERVL (Zhao et al., 2023b). Npm1 represses MERVL by regulating the protein stability of histone H1 variants. The interaction between Npm1 and histone H1 is regulated by the histone methyltransferase disruptor of telomeric silencing 1-like (Dot1l), which is co-enriched with Npm1 in MERVL (Zhao et al., 2023b). Therefore, Dot1l is also necessary for the repression of ERVs, including MERVL. Based on the above findings, it can be concluded that histone variants and chaperones actively contribute to the silencing of ERVs (Figure 3A and B).

### Chromatin remodelers

Upon the assembly of nucleosomes with histones, the spacing and mobility of nucleosomes along DNA are regulated by chromatin remodeling. Chromatin remodelers alter chromatin structure by regulating chromatin accessibility and nucleosome positioning and have been implicated in the repression of ERVs (Figure 3A and B). The Ep400–Tip60 (also known as Kat5) chromatin remodeling complex represses the ERV III family member MERVL in ESCs (Rodriguez-Terrones et al., 2018) while simultaneously contributing to the maintenance of ESC identity (Fazzio et al., 2008). Considering that Ep400–Tip60 can promote H3.3 deposition at promoters and enhancers (Pradhan et al., 2016), it is plausible to speculate that Ep400–Tip60 might employ H3.3 to suppress ERVs. The repressive activity of Ep400–Tip60 is bolstered by its interaction with the telomere protein Rap1 (Barry et al., 2022). Similar to Ep400, another chromatin remodeling factor Chd5, a member of the sucrose nonfermenting 2 (SNF2)-like family of ATPases, represses MERVL (Hayashi et al., 2016). Loss of *Chd5* leads to decreased level of H3K27me3 and increased loading of histone variants H3.1 and H3.2 at MERVL loci (Hayashi et al., 2016). While H3K27me3 is unlikely to regulate MERVL, Chd5 probably exerts control through H3.1 and H3.2. Another member of the SNF2-like family of ATPases involved in ERV regulation is Hells (also known as Lsh, Lsh1, PASG, or Smarca6). Hells interacts with Dmnt3a/b and maintains DNA methylation on IAP, SINE B1, and LINE1 in mouse tissues and embryos (Dennis et al., 2001; Huang et al., 2004). ESCs lacking *Hells* exhibit a reduced ability to silence MuLV vectors (Zhu et al., 2006). However, *Hells* depletion does not affect IAP expression in ESCs (Xi et al., 2009), suggesting the presence of other repressors to complement the function of Hells. Smarcad1, yet another chromatin remodeler, has been discovered to restrict ERVs (Figure 3A). Smarcad1 is recruited by Trim28 to silence class I and class II ERVs (Sachs et al., 2019). These studies highlight that chromatin remodelers are recruited by epigenetic regulators to further restrain ERVs in ESCs.

### Chromatin architecture

The nucleosome chains are further organized into three-dimensional (3D) structural domains. The 3D architecture of chromatin is associated with the regulation of ERVs in ESCs (Figure 3B). Comparatively, MERVL^+^ 2-cell-like cells display more relaxed 3D chromatin structures than ESCs (Zhu et al., 2021b). In addition, depletion of the insulator protein CCCTC-binding factor (CTCF) or disruption of the cohesin complex leads to disorganized 3D chromatin and activates MERVL in ESCs (Olbrich et al., 2021; Zhu et al., 2021b). Disruption of rRNA biogenesis also results in the reorganization of 3D chromatin around MERVL and elevates MERVL expression (Yu et al., 2021). Provided that CTCF binds to multiple TEs in mammalian cells (Bourque et al., 2008; Kunarso et al., 2010), it is worth examining whether other ERVs in ESCs are repressed by the 3D genome structure.

Currently, several epigenetic pathways have been implicated in the restriction of ERVs in ESCs. However, it remains unclear which regulatory pathway is the primary mechanism for controlling ERVs. Numerous epigenetic regulators have been linked to the control of MERVL, but the extent to which each pathway contributes to its repression is still uncertain. It is possible that disruption of one epigenetic pathway may alter other epigenetic marks.

## Post-transcriptional regulation and other regulatory factors of ERVs

### RNA modifications

Once transcribed, ERVs encounter blockage by post-transcriptional regulators. This phenomenon is exemplified by the case of MERVL. The paraspeckle-localized RNA-binding protein Pspc1 recruits Tet2, Hdac1, and Hdac2 to MERVL DNA through its interaction with chromatin-bound MERVL RNA. Subsequently, Tet2 catalyzes methylated MERVL RNA to acquire the 5hmC modification, which destabilizes MERVL RNA (Guallar et al., 2018). Alternatively, ERV RNA can be modified by *N*^6^-methyladenosine (m^6^A). m^6^A RNA modification is highly abundant in ERV III family members (e.g. MTA) and RLTR10 in oocytes (Wu et al., 2022). In 2-cell embryos, m^6^A is enriched in MERVL and ERVL-MaLR (e.g. ORR1A0 and ORR1A1). KIAA1429, a component of the m^6^A transferase complex, guarantees the timely decay of maternal mRNAs after ZGA and maintains the stability of MTA in oocytes (Wu et al., 2022). Mettl3 deposits m^6^A on MERVL during ZGA and mediates the degradation of MERVL after the 2-cell stage (Wu et al., 2022). Both the methyltransferases of m^6^A, Mettl3 and Mettl14, and the reader of m^6^A, Ythdc1, are required to silence ERVs in ESCs (Chelmicki et al., 2021; Chen et al., 2021a; Liu et al., 2021; Xu et al., 2021). Mettl3 and Mettl14 form a complex with Wtap and Zc3h13 to repress IAPs and other ERVKs (Chelmicki et al., 2021). Ythdc1 is recruited by m^6^A-modified ERV RNA and represses target ERVs, such as ERVKs and LINE1, through Trim28/Setdb1-mediated H3K9me3 (Chen et al., 2021a; Liu et al., 2021; Xu et al., 2021). Moreover, other RNA-binding proteins can recruit repressors to ERVs in a manner similar to Pspc1 and Ythdc1. Spen directly binds to ERVK transcripts and then recruits class I HDACs to repress the transcription of ERVK loci (Carter et al., 2020). These findings illustrate the ERV-suppressive role of RNA-binding proteins in ESCs and early embryos.

### Small RNAs

Small RNAs serve as another layer of defense against ERVs. One important group of ERV regulators are PIWI-interacting RNAs (piRNAs). piRNAs are derived from the cleavage of retrotransposon RNAs and were initially discovered to silence retrotransposons in the germline (Ernst et al., 2017). Recent evidence supports the presence of piRNAs in mouse preimplantation embryos, indicating their potential role in combating ERVs (Garcia-Lopez et al., 2014, 2015; Yang et al., 2016). Another group of small RNAs, called endo-siRNAs, can be generated from ERVs when acute DNA demethylation occurs in ESCs (Berrens et al., 2017). Deletion of factors critical for endo-siRNA production (Ago2 and Dicer) further activates IAPs and LINEs in ESCs (Berrens et al., 2017). Endo-siRNAs are crucial to the immediate repression of ERVs, whereas long-term silencing of ERVs depends on chromatin modifications (Berrens et al., 2017). Endo-siRNAs are also able to dampen the stability of ERV LTR-driven chimeric transcripts in mouse oocytes (Su et al., 2021). MicroRNAs (miRNAs), which differ from endo-siRNAs in that they rely on the Drosha–DGCR8 complex for biogenesis, indirectly regulate ERV expression by repressing other direct regulators such as Gata2 and Zmym2 (Choi et al., 2017; Yang et al., 2020). Depletion of *Dicer*, which is responsible for miRNA maturation, disrupts miRNA expression and activates MERVL in blastocysts (Ohnishi et al., 2010). Once Setdb1 and H3K9me3 are lost in ESCs, tRNA-derived small RNAs (tsRNAs) serve as another line of defense against ERVs. Specifically, 22-bp tsRNAs suppress ERV translation, whereas 18-bp tsRNAs interfere with the retrotransposition and replication of ERVs (Schorn et al., 2017).

### Cooperation between post-transcriptional and chromatin regulators

Post-transcriptional factors can cooperate with chromatin regulators to regulate ERVs. The RNA transcribed by LINE1 serves as a nuclear lncRNA that functions as a scaffold (Percharde et al., 2018). LINE1 lncRNA recruits nucleolin and Trim28 to repress rDNA and Dux (Percharde et al., 2018). Consequently, LINE1 RNA depletion indirectly activates MERVL expression. ERVs themselves also function as lncRNAs. Nuclear RNAs transcribed from IAPEz, MMERVK10C, MMERVK9C, and MMETn have the ability to sequester transcription condensates containing RNA polymerase II and the Mediator coactivators to ERV chromatin loci (Asimi et al., 2022). Overexpression of pluripotency transcription factors (Oct4, Sox2, Klf4, and Myc) competes with ERV RNAs for the recruitment of chromatin condensates, resulting in the downregulation of ERVs (Asimi et al., 2022). Similarly, depletion of ERV RNAs results in the suppression of the enhancer activity of ERV chromatin loci (Asimi et al., 2022). Protein kinases and kinase-catalyzed protein phosphorylation have also been implicated in the regulation of ERVs. Pim3 kinase indirectly suppresses ERVs (MERVL, LINE1, RTLR6, RLTR45-int, and ERVB4) through class IIa HDACs (Zhao et al., 2022). In the absence of Pim3, phosphorylated adenosine 5′ monophosphate-activated protein kinase (AMPK) mediates the phosphorylation and export of class IIa HDACs from the nucleus to the cytoplasm, thereby activating ERVs (Zhao et al., 2022). Inhibition of Pim3 kinase activity or activation of AMPK phosphorylation similarly activates MERVL, whereas the addition of an AMPK inhibitor can repress MERVL expression (Zhao et al., 2022).

### Other post-transcriptional regulators

There are additional regulators involved in the restriction of ERVs beyond transcription. The nuclear exosome targeting (NEXT) RNA degradation complex represses MERVL and MusD in ESCs, although its mechanism of action is independent of NEXT-based RNA decay (Wu et al., 2019). Loss of the RNA-binding protein Lin28 activates all classes of ERVs, particularly MERVL (Sun et al., 2021). The activation of MERVL stems from the upregulation of *Dux* and the release from the repression by Ncl/Trim28 upon *Lin28* deletion (Sun et al., 2021). Recent studies have linked the cell cycle to the activation of MERVL and the acquisition of a 2-cell-like state (Zhu et al., 2021a; Nakatani et al., 2022). ESCs that experience cell cycle arrest at G1/early S phase display diminished perinucleolar heterochromatin, which permits the expression of *Dux*, leading to the activation of MERVL and the adoption of a 2-cell-like state (Zhu et al., 2021a). It has been discovered that reducing replication fork speed induces MERVL in the S phase (Nakatani et al., 2022). Taken together, the above examples demonstrate the possibility of repressing ERVs beyond transcription.

## Conclusions and future perspectives

**Figure 4:**
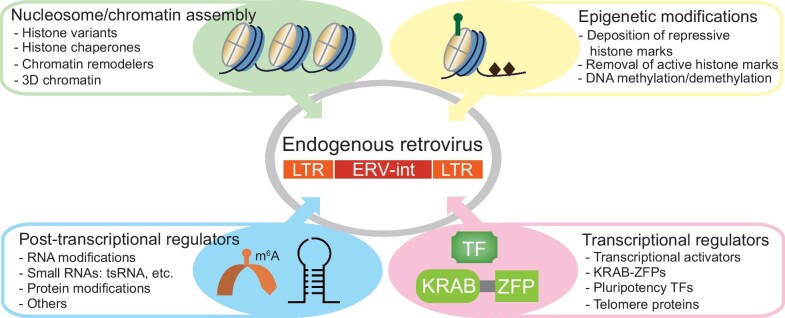
Modulation of ERVs by transcriptional regulators, nucleosome/chromatin assembly, epigenetic modifications, and post-transcriptional regulators. TF, transcription factor.

This review comprehensively summarizes diverse strategies to regulate ERVs in mouse ESCs and early embryos (Figure 4). ERVs are regulated at multiple interconnected levels. To repress ERVs, some silencing strategies are used when others fail, as exemplified by the Dmnt1–Setdb1–tsRNA axis. Setdb1 is able to safeguard ERVK in the absence of *Dmnt1* and *Uhrf1* (Sharif et al., 2016). In the absence of Setdb1, tsRNAs repress ERV translation (Schorn et al., 2017). After the removal of repression barriers, the successful activation of ERV expression relies on transcription factors recognizing ERVs, as evidenced by various indirect stimulations of transcription leading to ERV activation (Figure 1A). In support of the key role of transcription in ERV activation, depletion of key transcriptional activators, such as Dux, can partially restore the repression of ERVs in the absence of ERV repressors (Yang et al., 2020). Furthermore, overexpression of key transcriptional activators is sufficient to activate ERV expression in the presence of ERV repressors (Figure 1A). Our current understanding of ERV regulation is mostly based on studying ERV subfamilies as a whole due to high sequence similarity within the same ERV subfamily. However, distinct ERV loci can display differential regulation depending on the chromatin context. Additionally, each ERV locus may have acquired unique mutations and lost specific sequences during evolution. It remains a challenge to discriminate between different ERV loci. A thorough comprehension of ERV regulation will not only enhance our understanding of these repeat elements but also shed light on the regulatory mechanisms of other TEs or repetitive sequences within the genome.

## Notes

**Author contributions:** X.L. drafted the manuscript text and prepared figures.
